# Postoperative Patient-Reported Outcomes after Uniportal Video-Assisted Thoracoscopic Surgery Using the Perioperative Symptom Assessment for Lung Surgery Scale

**DOI:** 10.3390/curroncol29100604

**Published:** 2022-10-13

**Authors:** Ding Yang, Qian Hong, Chenguang Zhao, Juwei Mu

**Affiliations:** Department of Thoracic Surgery, National Cancer Center/National Clinical Research Center for Cancer/Cancer Hospital, Chinese Academy of Medical Sciences and Peking Union Medical College, Beijing 100021, China

**Keywords:** lung surgery, patient-reported outcomes, postoperative symptoms, VATS

## Abstract

This study aimed to use a new special inventory for lung surgery patients to evaluate postoperative symptoms and functional status and to identify factors that may affect these after uniportal video-assisted thoracoscopic surgery (VATS). In this single-center longitudinal cohort observational study, we used a new scale, the perioperative symptom assessment for lung surgery (PSA-Lung), to evaluate the recovery from symptoms and the functional status of patients undergoing uniportal VATS. We divided patients into two groups, according to patients’ symptom scores, and compared the clinical characteristics between the two groups under each item. Then, we conducted a qualitative interview regarding coughing in postoperative week 4. Exactly 104 patients were enrolled in this study. The two highest-scoring patient-reported outcome (PRO) items were “shortness of breath” and “coughing” in the fourth week after surgery. Thirty-one patients reported that “coughing” severely influenced their lives in postoperative week 4. Using the PSA-Lung inventory, we found that “shortness of breath” was the worst symptom in postoperative week 4. Although “coughing” was not the most important symptom in the early postoperative period, it affected some patients’ lives in postoperative week 4. Therefore, further research is required to determine the optimal cut-off point for coughing.

## 1. Introduction

Surgeons are constantly improving their surgical techniques to achieve positive outcomes in minimally invasive thoracic surgery, where the aim is completing surgical operations with minimal trauma. Video-assisted thoracoscopic surgery (VATS) has been carried out extensively worldwide, and traditional thoracotomy can be avoided in almost all lung surgeries [1,2,3]. When VATS is performed with minimal invasion in the thoracic wall, avoiding the severe neuromuscular trauma associated with traditional thoracotomy, this can significantly enhance the speed of recovery, improve the patient’s postoperative quality of life, and reduce postoperative complications [4,5]. One previous study has confirmed that the postoperative symptom burden of VATS is less than that of thoracotomy [6].

The clinical outcomes of uniportal VATS are comparable to multiportal VATS but have more advantages in reducing postoperative pain and chest wall paresthesias [7,8,9,10,11]. Another study has shown that uniportal VATS produces fewer severe symptoms and a better functional status than multiportal VATS [12].

Patient-reported outcomes (PROs) have recently received increasing attention from clinicians and researchers. PROs can be employed for proactive symptom management, to evaluate the effectiveness of different surgical treatment plans, and to facilitate the implementation of shared decision-making between doctors and patients [13,14,15,16]. Therefore, the characteristics of postoperative PRO changes in patients undergoing lung surgery and the associated influencing factors are also receiving increasing attention.

In fact, perioperative symptom assessment has been implemented in clinical practice; PRO-based symptom management decreased symptom burden and the incidence of complications compared with usual care, which was confirmed by a multicenter RCT [17]. Another study also showed that combining PROs with routine care could increase survival better than usual care for patients with metastatic cancer [13]. PROs also can be used in predicting post-discharge complications for lung cancer surgery patients [18].

There are many PRO scales for lung cancer [19]. One of the most widely used inventories is the MD Anderson Symptom Inventory-Lung Cancer (MDASI-LC)-specific scale. Many previous studies have used MDASI-LC to assess the symptom burden and functional recovery of perioperative lung surgery patients [20,21,22]. However, MDASI-LC was designed for patients undergoing chemotherapy and radiotherapy, and no perioperative-specific symptoms or functional items were included. When the scale was developed, minimally invasive thoracic surgery was not as widely used as it is today.

This study aimed to use a new special inventory for lung surgery patients to evaluate postoperative symptoms and functional status and to identify factors that may affect these.

## 2. Materials and Methods

### 2.1. Study Participants

This was a single-center longitudinal cohort observational study conducted from December 2021 to June 2022 at the Cancer Hospital, Chinese Academy of Medical Sciences. The inclusion criteria were as follows: (1) age greater than or equal to 18 years; (2) planned uniportal video-assisted thoracoscopic lung surgery; and (3) the ability to understand the content and purpose of the study. The exclusion criteria were as follows: (1) postoperative complications during hospitalization; (2) unplanned reoperation; (3) incomplete perioperative data; (4) serious heart, brain, and liver comorbidities, or other serious diseases; and (5) patients receiving preoperative or induction treatments. The patients in our study were operated on by same surgical treatment team.

This study was approved by the Ethics Committee of National Cancer Center/Cancer Hospital, Chinese Academy of Medical Sciences, and Peking Union Medical College (Approval No. 22/301-3503). Patient consent was obtained at the time of enrollment.

### 2.2. Patient-Reported Outcome Measurements and Data Collection

Our primary outcomes were symptom severity and functional status after uniportal VATS lung surgery based on PROs. The Perioperative Symptom Assessment for Lung Surgery (PSA-Lung) was used for PRO assessments. The PSA-Lung scale includes seven symptom items (pain, coughing, shortness of breath, disturbed sleep, fatigue, drowsiness, and distress) and two functional items (interference of activity and walking). Each symptom’s severity was rated between 0 (the absence of symptom) and 10 (the worst imaginable symptom). Similarly, functional items were also rated on a scale between 0 (no interference) and 10 (complete interference). The PSA-Lung scale development team has verified its reliability and validity in lung cancer surgery patients, and the research results suggested adequate reliability and validity. The relevant articles have been submitted for publishing, and the preliminary results were announced at the 28th Annual Conference of the International Society for Quality of Life Research [23].

All patients were evaluated using PSA-Lung as a baseline before surgery; afterwards, they were assessed on postoperative days (PODs) 1–4 and every week until the fourth week. During hospitalization, the researchers asked the patients to score each item on the scale at their bedside, and the scales were returned simultaneously. After discharge, the researchers conducted further questionnaire interviews with the patients by telephone or instant messenger.

According to NCCN Guidelines, a score of 4 or greater is usually defined as moderate or severe for adults and may indicate the need for additional clinical attention [24,25,26]. Dai Wei et al. defined ≥4 on a 0–10 scale as a threshold symptom score after lung cancer surgery and stated that clinicians would respond when PRO scores reached the threshold. Their study showed the advantages and feasibility of PRO-based perioperative management [17]. Therefore, we divided patients under each item into the following two groups according to the patients’ symptom scores in the fourth week: 0–3 as good recovery, and >3 as poor recovery. We compared clinical characteristics (age, gender, body mass index (BMI), smoking index, education level, FEV1 (forced expiratory volume in one second), FEV1%, FEV1%FVC (forced vital capacity), MVV (maximal voluntary ventilation), MVV%, DLCO SB (diffusion capacity of the lungs for carbon monoxide, single-breath), DLCO SB%, tumor pathologic stage, tumor histologic type, extent of the procedure, type of lymphadenectomy, and operation time) between the two groups under each item.

### 2.3. Interview Regarding Coughing

In the preliminary clinical observation, we discovered that the cough symptom of lung surgery patients worsened after discharge and was the most common symptom that patients complained about in the fourth follow-up week. Therefore, we conducted an additional simple qualitative interview for each patient over telephone during the fourth follow-up week. The interviews were qualitative, one-to-one interviews including the following 3 questions: (1) Does coughing affect your daily life? (2) What do you think the current severity of the coughs is? No, mild or severe? (3) Do you have any other feelings about coughing that you wish to tell us? Each interview lasted for 3–5 min per patient. All the interview content was recorded for further analysis.

### 2.4. Statistical Analyses

Available PRO data for the nine time points were included in the analyses, including pre-operation, PODs 1–4, and post-discharge weeks 1–4. The t-test was used for normally distributed continuous variables, and the Mann–Whitney U test was used for abnormal distribution data. Chi-square tests and Fisher’s exact test were used for categorical variables. The statistical analyses were 2-tailed, and *p*-value < 0.05 was considered statistically significant. All the statistical analyses were performed with SPSS version 22.0.

## 3. Results

### 3.1. Patients’ Characteristics

A total of 104 patients (44 males and 60 females) were consecutively enrolled in this study. The median age was 57 years. All patients were prepped for uniportal VATS, and two underwent intraoperative conversion to open procedure. The median length of postoperative hospital stay was 4 days. The enhanced recovery after surgery (ERAS) perioperative care pathway was not used, and all patients were managed with usual care. Baseline clinical characteristics of patients are shown in Table 1.

### 3.2. Patient-Reported Outcome Characteristics

**Figure 1:**
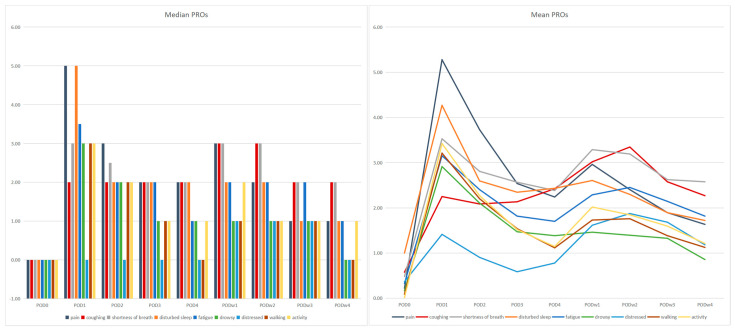
The median and mean values of each PRO score (pain, coughing, shortness of breath, disturbed sleep, fatigue, drowsy, distressed, activity, walking) at each data collection time point.

**Figure 2:**
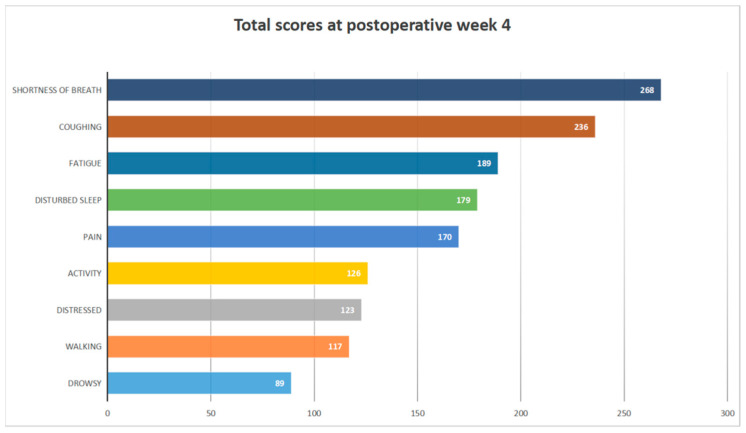
Total scores at postoperative week 4.

We calculated the median and mean values of each PRO score (pain, coughing, shortness of breath, disturbed sleep, fatigue, drowsiness, distress, activity, and walking) at each data collection time point, as shown in Figure 1. The graphs show the changes in each PRO score with time postoperatively. The PRO scores rose and fell after discharge, and most PROs failed to return to their preoperative baseline levels in the fourth postoperative week, both at the median and mean values. The two highest-scoring PRO items in week 4 after surgery were “shortness of breath” (median 2, mean 2.58) and “coughing” (median 2, mean 2.27). To make the results of the fourth week more intuitive, we summed the scores of the 104 patients under each item at week 4 after surgery, as shown in Figure 2, and we discovered that “shortness of breath” and “coughing” had the highest overall scores.

Detailed, comparative results of the good and poor recovery groups are shown in Table 1. We discovered that the preoperative pulmonary ventilation function is highly important for uniportal VATS postoperative symptoms and functional recovery. There were statistically significant differences in the FEV1% and MVV% values between the two groups under all PRO items; however, DLCO SB and DLCO SB% appeared to have little effect on the recovery of patients.

### 3.3. Interview Regarding Coughing

Forty-six patients (median coughing score = 2) reported that coughing did not affect their current lives. Twenty-seven patients reported mild influence (median coughing score = 2), and thirty-one (median coughing score = 3) reported severe influence.

## 4. Discussion

The PSA-Lung is essentially a new Patient-Reported Outcome-based scale, which has been designed for lung surgery patients and differs from the past perioperative evaluation indicators, such as chest X-rays, laboratory tests, days of drainage tube indwelling, postoperative drainage, and postoperative hospital stay. We could establish PRO-based perioperative symptom management, evaluate the benefits and drawbacks of different lung surgeries, and analyze trends in patients’ postoperative symptoms and functional recovery with the use of PSA-Lung. Additionally, PSA can allow surgeons and patients to understand symptoms and functions based on data, which may improve shared decision-making [27].

Currently, minimally invasive procedures and ERAS are widely accepted by surgeons and patients, and VATS has become the mainstay treatment for early-stage lung cancer surgery [28,29,30]. NCCN Guidelines Version 4.2022 Non-Small Cell Lung Cancer recommend that VATS or minimally invasive surgery (including robotic-assisted approaches) should be strongly considered for patients with no anatomic or surgical contraindications, as long as there is no compromise of the standard oncologic and dissection principles of thoracic surgery.

Past studies demonstrated that patients with locally advanced lung cancer who received VATS experienced a lower symptom burden and less daily function interference than those who underwent thoracotomy [6], and uniportal VATS may result in fewer severe symptoms and a better functional status than multiportal VATS for lung cancer [12]. Therefore, this study focused on uniportal VATS. We did not perform uniportal VATS on all lung surgery patients. If the lesion diameter was larger than five centimeters and invasion in vital blood vessels or bronchus, serious thoracic cavity adhesion, accidental intraoperative bleeding, or other complex operations were required, we selected or changed to thoracotomy.

Our center has been proficient in performing uniportal VATS for over 7 years [31]. This observational study described PRO changes 4 weeks after uniportal VATS and analyzed the relationship between PROs and pulmonary function.

In previous studies, pain has often been reported as the most severe symptom, with the slowest recovery in patients after lung surgery [6,32,33]. According to the trend of postoperative PROs in our study, we discovered that pain scores were highest (median 5, mean 5.28) on POD 1 after which they gradually decreased. On POD 4, the median and mean dropped to 2 and 2.24, respectively. By the fourth week, most patients (*n* = 94, 90.38%) scored 0-3 points, the median was 1, and the mean was 1.63, suggesting that most patients experienced mild to no pain. Therefore, this shows the significance of uniportal VATS in reducing postoperative pain in patients.

Moreover, we observed that in the early postoperative period, coughing was not a severe symptom for most patients. Nevertheless, in the second postoperative week, the coughing score increased significantly and had the highest PRO score. In the fourth postoperative week, the coughing score decreased. Only 20 (19.2%) patients scored ≥4; however, by conducting interviews, we discovered that 31 (28.9%) patients still felt that coughing was seriously affecting their lives, and these patients’ median coughing score was 3. The definition of coughing recovery requires further exploration.

The impact of preoperative pulmonary function on postoperative PROs is another concern that requires our attention. Shortness of breath is closely related to pulmonary function. It had the highest overall and mean scores in the fourth week. Before surgery, surgeons usually determine whether the patient can tolerate lung surgery by assessing the patient’s pulmonary function. The current, widely accepted evaluation metrics of pulmonary function are as follows: (1) pneumonectomy: FEV1 > 2 L, MVV% > 50%; (2) lobectomy: FEV1 > 1 L, MVV% > 40%; (3) segmentectomy and wedge resection: FEV1 > 0.6, MVV% > 35% [34]. Nevertheless, other researchers believe that FEV1% is a more sensitive and individual index to predict postoperative survival and postoperative complications, and a preoperative FEV1% < 60% was a strong predictor for respiratory complications [35,36,37]. Our study further clarified the importance of pulmonary function, especially FEV1% and MVV%, for the postoperative rehabilitation of patients. Moreover, in patients with good pulmonary function (FEV1%, mean, 101.23; MVV%, mean 83.31), higher FEV1% and MVV% scored may indicate a lower symptom burden and good functional recovery in patients in the fourth week after lung surgery. Previous studies also showed that breathing exercises could improve pulmonary function, decrease the incidence of postoperative pulmonary complications, and decrease the length of hospital stay after lung cancer surgery [38]. Therefore, a routine preoperative pulmonary function exercise needs to be undertaken by all patients, and it is highly important for postoperative recovery from symptoms and functional status.

Moreover, there are still some limitations in our study. First, since the enrolled patients predominantly had early-stage tumors; therefore, the trend of symptom changes in the study may not be representative of patients with advanced-stage tumors. Second, this was a single-center study, and the results may have been biased. Therefore, the evaluation and outcomes from this study still require more multi-center studies to verify them. Finally, the factors affecting postoperative cough seemed to be related only to preoperative lung function. This may be related to the fact that most of our enrolled patients had early-stage lung cancer; these patients often have the same anesthesia procedure, similar surgical procedure, postoperative medication, and postoperative care. Therefore, we simply suggest that preoperative lung function exercises should be essential for all lung surgery patients. We did not perform any interventions on the enrolled patients because this was an observational study. Whether medication or other preventive measures are effective for coughing requires a new randomized controlled trial.

## 5. Conclusions

Using the PSA-Lung inventory, we identified that “shortness of breath” was the worst symptom in postoperative week 4, whereas the “pain” score was lower, showing that patients recovered faster from pain after uniportal VATS. Although “coughing” was not the most important symptom in the early postoperative period, its score rose and peaked after discharge, which affected patients’ lives in postoperative week 4. Therefore, further research is required to determine the optimal cut-off point for coughing. Finally, FEV1% and MVV% are important factors affecting patient’s postoperative symptoms and functional recovery.

## Figures and Tables

**Table 1:** Clinical Characteristics and PROs in fourth week after surgery.

Variable	Total	Pain		*p* Value	Cough		*p* Value	SOB		*p* Value	DS		*p* Value		
		0–3	>3		0–3	>3		0–3	>3		0–3	>3			
Patients (number)	104	94	10	/	84	20	/	82	22	/	90	14	/		
Age (year), median	57	57	62.5	0.11	57	60	0.52	56	60.5	0.05	57	62.5	0.34		
Female/male	60/44	54/40	4/6	1	51/33	9/11	0.22	48/32	12/10	0.81	52/38	8/6	1		
BMI, mean	23.83	23.73	24.81	0.48	23.86	23.75	0.98	23.69	24.38	0.38	23.77	24.26	0.33		
Smoking index, median	0	0	0	0	0	0	0	0	0	1	0	0	1		
Education level				0.19			0.78			0.64			0.77		
Middle school or below	61	53	8		53	8		47	14		52	9			
Above middle school	43	41	2		31	12		35	8		38	5			
FEV1, median	2.63	2.66	2.11	0.01	2.64	2.39	0.38	2.64	2.47	0.15	2.64	2.18	0.02		
FEV1%, mean	101.23	102.81	86.39	0.01	104.09	89.20	0.00	103.46	92.92	0.02	103.46	86.90	0.00		
FEV1%FVC, mean	79.97	80.37	76.20	0.13	79.96	79.99	0.77	80.29	78.76	0.51	80.50	76.52	0.13		
MVV, mean	86.76	89.99	56.38	0.00	88.26	80.44	0.17	89.30	77.30	0.08	89.29	70.52	0.03		
MVV%, mean	83.31	86.15	56.53	0.00	85.83	72.72	0.01	85.73	74.27	0.03	85.38	69.96	0.04		
DLCO SB, median	7.82	7.92	7.08	0.14	7.88	7.60	0.73	7.82	7.79	0.63	7.88	7.60	0.71		
DLCO SB%, mean	94.34	94.98	88.31	0.24	95.35	90.12	0.13	94.31	94.46	0.77	94.81	91.29	0.51		
Tumor pathologic stage				0.46			0.90			0.70			0.30		
Tis	15	15	0		12	3		12	3		14	1			
I	64	57	7		52	12		49	15		57	7			
II	4	3	1		3	1		3	1		3	1			
III	3	3	0		2	1		2	1		2	1			
Benign or other tumor	18	16	2		15	3		16	2		14	4			
Tumor histologic type				0.28			0.15			1.00			0.51		
Adenocarcinoma	77	71	6		65	12		61	16		68	9			
Non-adenocarcinoma	27	23	4		19	8		21	6		22	5			
Extent of the procedure				0.55			0.29			0.58			0.46		
Pneumonectomy	1	1	0		0	1		1	0		1	0			
Lobectomy	39	34	5		32	7		29	10		32	7			
Sub-lobar	64	59	5		52	12		52	12		57	7			
Type of lymphadenectomy				1.00			0.79			0.81			1.00		
Systematic dissection	92	83	9		73	19		71	21		79	13			
Sampling	10	9	1		9	1		9	1		9	1			
Not performed	2	2	0		2	0		2	0		2	0			
Operation time(minute), median	106	106.5	116	0.93	107	104.5	0.64	106.5	106	0.80	105.5	119	0.41		
Variable	fatigue		*p* value	drowsy		*p* value	distressed		*p* value	walking		*p* value	activity		*p* value
	0–3	>3		0–3	>3		0–3	>3		0–3	>3		0–3	>3	
Patients (number)	89	15	/	99	5	/	93	11	/	94	10	/	96	8	/
Age (year), median	56	65	0.00	57	69	0.01	57	64	0.04	56.5	66	0.01	57	66	0.02
Female/male	54/37	6/9	0.163	60/39	0/5	0.012	56/37	4/7	0.196	55/39	5/5	0.74	56/40	4/4	0.719
BMI, mean	23.69	24.68	0.25	23.86	23.29	0.84	23.80	24.10	0.65	23.86	23.64	0.96	23.79	24.33	0.40
Smoking index, median	0	200	0	0	900	0	0	300	0	0	150	0	0	150	0
Education level			0.09			1.00			0.76			0.74			0.72
Middle school or below	49	12		58	3		55	6		56	5		57	4	
Above middle school	40	3		41	2		38	5		38	5		39	4	
FEV1, median	2.64	2.18	0.05	2.63	1.98	0.09	2.64	2.04	0.01	2.66	2.01	0.00	2.64	2.01	0.00
FEV1%, mean	103.57	87.32	0.00	102.48	76.38	0.01	103.88	78.76	0.00	103.49	79.96	0.00	103.09	78.90	0.00
FEV1%FVC, mean	80.62	76.07	0.02	80.36	72.09	0.04	80.59	74.69	0.05	80.42	75.66	0.09	80.36	75.28	0.60
MVV, mean	90.24	66.11	0.00	88.09	60.46	0.04	89.88	60.38	0.00	90.04	55.89	0.00	89.65	52.10	0.00
MVV%, mean	86.71	63.11	0.00	84.70	55.78	0.02	86.48	56.50	0.00	86.26	55.58	0.00	86.00	51.04	0.00
DLCO SB, median	7.90	7.53	0.76	7.90	6.19	0.09	7.90	7.48	0.84	7.91	6.65	0.30	7.88	7.09	0.74
DLCO SB%, mean	94.84	91.37	0.23	95.28	75.74	0.03	94.84	90.11	0.19	95.08	87.37	0.06	94.60	91.28	0.21
Tumor pathologic stage			0.07			1.00			0.68			0.65			0.63
Tis	15	0		15	0		14	1		13	2		14	1	
I	54	10		60	4		56	8		28	6		59	5	
II	2	2		4	0		3	1		3	1		3	1	
III	2	1		3	0		3	0		3	0		3	0	
Benign or other tumor	16	2		17	1		17	1		17	1		17	1	
Tumor histologic type			0.21			0.11			0.15			0.28			0.43
Adenocarcinoma	68	9		75	2		71	6		71	6		72	5	
Non-adenocarcinoma	21	6		24	3		22	5		23	4		24	3	
Extent of the procedure			0.36			0.40			0.40			0.11			0.12
Pneumonectomy	1	0		1	0		1	0		1	0		1	0	
Lobectomy	31	8		36	3		33	6		32	7		33	6	
Sub-lobar	57	7		62	2		59	5		61	3		62	2	
Type of lymphadenectomy			1.00			1.00			0.68			0.67			1.00
Systematic dissection	78	14		87	5		81	11		82	10		84	8	
Sampling	9	1		10	0		10	0		10	0		10	0	
Not performed	2	0		2	0		2	0		2	0		2	0	
Operation time (minute), median	107	101	0.89	107	101	0.93	105	127	0.37	105	143	0.05	105	143	0.08

## Data Availability

The data is presented in this study are available in this article.
